# Medical students’ knowledge of race-related history reveals areas for improvement in achieving health equity

**DOI:** 10.1186/s12909-022-03650-x

**Published:** 2022-08-10

**Authors:** Charles Sanky, Halbert Bai, Celestine He, Jacob M. Appel

**Affiliations:** 1 Department of Medical Education, Icahn School of Medicine at Mount Sinai, New York, NY USA; 2 Department of Emergency Medicine, Icahn School of Medicine at Mount Sinai, New York, NY USA; 3 Department of Psychiatry, Icahn School of Medicine at Mount Sinai, New York, NY USA

**Keywords:** Health equity, Race, Medical history, Medical education, Anti-racism

## Abstract

**Background:**

Medical schools have increasingly integrated social justice, anti-racism, and health equity training into their curricula. Yet, no research examines whether medical students understand the complex history of racial injustice. We sought to investigate the relationship between medical students’ historical knowledge and their perceptions regarding health equity.

**Methods:**

Medical students at one large urban medical school self-rated their familiarity and importance of various racially-significant historical events and persons, as well as their agreement with statements regarding health equity, education, and preparedness to act. Descriptive and multivariate analyses were conducted in R.

**Results:**

Of 166 (RR=31.3%) participants, 96% agreed that understanding historical context is necessary in medicine; yet 65% of students could not describe the historical significance of racial events or persons. Only 57% felt that they understood this context, and the same percentage felt other medical students did not. A minority of students felt empowered (40%) or prepared (31%) to take action when they witness racial injustice in healthcare. Multiracial identity was significantly associated with increased knowledge of African American history (*p*<0.01), and a humanities background was significantly associated with increased knowledge of Latin American history (*p*=0.017). There was a positive, significant relationship between advocacy statements, such as “I have taken action” (*p*<0.001) and “I know the roots of racism” (*p*<0.001) with mean familiarity of historical events.

**Conclusions:**

This study demonstrates that while students agree that racism has no place in healthcare, there remains a paucity of knowledge regarding many events and figures in the history of American race relations and civil rights, with implications for future physicians’ patient care and health equity efforts.

**Supplementary Information:**

The online version contains supplementary material available at 10.1186/s12909-022-03650-x.

## Background

Structural racism is a significant factor in healthcare in the United States. People from historically marginalized racial and ethnic groups, especially Black and Latino patients, are often treated negatively, both inside and outside of medical systems, in ways that adversely impact health. Within direct patient care, physicians continue to use race-based calculations for diagnostic tests [1–4], teach race as risk factors for disease instead of considering social determinants of health [5], and hold biases when making medical decisions [6]. Systemic racism further exacerbates health inequity by underfunding hospitals that primarily treat historically marginalized populations [7, 8] and creating wealth disparities that negatively influence social factors, such as housing instability, environmental health, and food insecurity, that are key determinants of health outcomes [7, 9]. In New York City, where our institution is located, structural racism has led to Black non-Latino maternal pregnancy-related mortality rate to be eight times higher than for White non-Latino women [10], and for COVID-19 related deaths in 2020 to occur at higher rates in historically marginalized populations [7, 9]. Racist policies inside and outside of medical interactions foster inequitable health outcomes for people from historically marginalized racial and ethnic groups, and significant structural change is required to achieve health equity. Furthermore, the history of racism and societal oppression is intimately intertwined with the systemic factors that result in inequitable health outcomes. False racial hierarchies that have been reinforced throughout our history have given way to the continued oppression of people from historically marginalized communities [11]. Principles of weathering [12] and allostatic load [13] highlight how the accumulation of racial stress, sociopolitical marginalization, and discrimination further health disparities. These demonstrates how a racialized lived experience is deeply rooted in chronic, persistent, and historical examples of racial oppression. To this day, there are still persistent manifestations of our racial history, including differential access to care, mortality, morbidity, pain management, and other health disparities.

In acknowledgment of the impact of racism in health outcomes, there has been a growing effort to address racism in medical education. The belief is that by educating the next generation of medical trainees, future healthcare professionals and leaders can continue to combat systems of oppression. Medical schools have promoted initiatives to address racism and bias within the hospital system [14], started anti-racism book clubs [15], included patient-focused learning with a focus on social determinants of health in preclinical years [16], and committed to including anti-racism in the curriculum [14, 17]. To address long-standing racism in the clinical sphere, students have pushed to stop using race-based calculations in diagnostic tests, such as eGFR, encouraged administrators to promote health equity, and advocated to integrate the history of scientific racism into the curriculum [17, 18]. Recently, medical schools have joined together in creating a coalition, the Anti-Racist Transformation in Medical Education, which seeks to collaborate in effecting systemic change [19] and in March of 2020, the Liaison Committee on Medical Education (LCME), the accrediting body for medical schools in the U.S. and Canada, mandated that curricular content must include addressing biases in order to effectively provide care for a diverse society [20]. This mandate institutes an ethos that aims to inculcate egalitarian values and extinguish personal biases. The integration of anti-racism in medical school curricula is a significant first step towards reducing racism in medicine. However, while medical students are taught what it means to be anti-racist, the historical underpinnings of racial and societal injustice in the U.S. are often lacking in such innovative educational efforts, which may, in turn, impact further learning, integration, and practice of health equity.

The historical knowledge base of medical students is dependent on an array of curricular and extracurricular learning experiences deeply embedded in a students’ identities, previous schooling, and level of engagement with historical content and pedagogy. Intergroup relation theory suggests that increased knowledge and contact with an out-group reduces prejudice by revealing similarities and fostering connection [21–23]. Previous studies have demonstrated that greater factual knowledge about the history or culture of a different racial group can reduce negative attitudes towards them [24]. Similarly, social psychologists have found that increased content-based knowledge of others reduces discomfort and uncertainty in interactions with other groups, thereby decreasing avoidance of outgroups [25, 26]. Content-based knowledge reduces biases through de-categorization, undermining outgroup stereotypes and reducing intergroup bias [26]. Thus, integrating historical, context-based knowledge of racial history can help advance the social justice and health equity curricula of medical schools. Indeed, ignorance of racial history could lead to harmful consequences, such as perpetuating inequitable health outcomes, teaching race-based medicine, equating racial heritage with genetics [27], misdirection of physician advocacy, a lack of cultural humility, inducing racial trauma in patient communities and among peers, and even the inadvertent perpetuation of structural racism by individuals leading academic medical institutions [28].

While medical schools have increasingly integrated social justice, anti-racism, and health equity training into their curricula, there remains no research on the extent to which medical students understand the complex, nuanced history of different racial minorities and the injustices they have faced. This study hypothesizes there is a great discordance between students’ perspectives regarding the importance of health equity and anti-racism in medicine and students’ knowledge of racially significant historical events, resulting in profound implications for medical education, patient care, and commitment to and practice of health equity.

## Methods

### Sample

Participants included medical students enrolled at one medical school situated within a large academic medical center. Enrollment includes approximately 130 medical students per class. Our institution is located on the border of the Upper East Side and East Harlem, straddling two neighborhoods with the highest and lowest socioeconomical status in New York City. The health disparities among these communities are profound; estimated life expectancy alone is now estimated at eleven years longer for residents of the Upper East Side as compared to residents of East Harlem [29]. Approximately 84.3% of our patient population identifies as Black or Latino [30]. As such, our medical school has invested substantially in health equity through efforts such as a robust Office of Diversity and Inclusion, the development of community partnerships and outreach efforts, the formation of an Institute for Health Equity, and a Racism and Bias Initiative [31]. In particular, our Department of Medical Education has set a vision for an anti-racist curriculum, which has since been integrated throughout all four years of medical school in each course, lecture, and small group discussion. This has also translated to explicit anti-racism learning in the forms of journal clubs, events, health equity research funding, and opportunities to engage with and contribute to historically underserved communities.

Select students were designated from each class to serve as champions and assist in study recruitment via email listservs and class group messages containing an anonymous link via the Survey Monkey platform. Student responses were anonymous, except for demographic parameters collected. All students had received the same formal curriculum regarding race and bias, including orientation sessions, small group sessions, journal clubs, lectures, and courses dedicated to understanding social determinants of health and health equity, by the time of survey completion.

#### Survey Instrument

We designed an exploratory survey to assess knowledge of historical terms and perspectives regarding health equity and racism. The survey (Supplement A) was developed by consensus in collaboration with our institution’s diversity and inclusion leadership team, the committee of faculty, staff, and students overseeing the Racism and Bias Initiative [31], and our department of medical education. Questions were informed by and modeled after validated surveys examining medical students’ perspectives on care for underserved populations [32–34]. Our study was deemed exempt by the Institutional Review Board and approved by the medical school and student council.

The first part of the survey asked participants to self-rate how familiar they were with various racially-significant historical events. Three widely-used high school history textbooks were consulted, and twenty-five subjects related to the history of American race relations that were covered significantly in all three were selected for potential inclusion in the survey. The study team then narrowed these items down to reflect a balance among racial and ethnic groups and chronicity. The Stonewall Uprising, a pivotal moment in LGBTQIA history, was included for intersectionality of racial history with the history of other marginalized populations. Similarly, redlining and gentrification are processes that contribute to health inequity were included; these trends have affected multiple populations throughout American history. There are many terms and key figures directly related to race and oppression within medicine, such as Henrietta Lacks, Tuskegee, and James Marion Sims. However, these terms are inherently discussed throughout medical school curricula and reflect the expressions of such history in medicine, not the historical roots of racism and oppression themselves. Our study focuses on whether students understand the complex, nuanced history of different racial minorities and the injustices they have faced. Thus, testing for recall of salient examples emphasized in medical school would not adequately reflect actual understanding of systems of racial oppression and the structural consequences for historically marginalized communities.

To control for participants selecting that they were familiar with all terms or no terms, Martin Luther King Jr. was used as a positive control while Clyde R. Hoey was used as a negative control. We perceived Dr. King as ubiquitous, and if participants did not know about him, they were unlikely to know other examples of key racial justice-related figures and terms in American history. Conversely, Clyde Hoey was a U.S. Senator, Governor of North Carolina, and segregationist with problematic viewpoints. He is much more obscure, and if participants were familiar with him, they were likely to know other terms. Thus, these terms served as our positive and negative controls to ensure quality of our responses.

The second part of the survey asked participants to self-rate their agreement with advocacy statements regarding health equity, education, and taking action against racism in medicine. Finally, participants self-identified their gender, race, ethnicity, year in medical school, and college field(s) of study. At the end of the survey, participants were given a phone number for 24/7 crisis support, in case participants required support or experienced disturbing associations from completing the survey. Additionally, they were directed to our institutional Racism and Bias website for more information regarding current efforts.

#### Statistical analysis

The research team conducted descriptive analyses to examine the distribution, central tendencies, normality, and other characteristics of the data. Aggregate percentages, means, and standard deviations for all historical terms, belief statements, and demographic factors were characterized. College fields of study were qualitatively coded into three categories: Science, Technology, Engineering, and Math (STEM), humanities, or both. After testing for normality, multivariate analyses were conducted to make comparisons among demographics, knowledge, and belief statements where appropriate, informed by the study hypothesis. All analyses were performed using Microsoft Excel and R [35].

## Results

### Sample

Out of 531 medical students eligible to complete the survey, 166 students (31.3%) responded Table 1. 54 (32.53%) respondents were first-year medical students (MS1), 40 (24.1%) second-year medical students (MS2), 40 (24.1%) third-year medical students (MS3), and 27 (16.3%) fourth-year medical students (MS4). PhD students were grouped with the MS2 sample and scholarly year students were included with the MS3 sample in all subsequent analyses. A total of 107 (64.5%) students were STEM college majors, 31 (18.7%) were humanities majors, and 13 (7.8%) were both. 84 (50.6%) were women. 77 (46.39%) identified as White only, 40 (21.69%) as Asian only, 17 (10.24%) as Black/African American only, 6 (3.61%) as Latino only, and 21 (12.65%) as multi-racial. These demographics largely mirror AAMC demographics data for U.S. medical students [36]. There were no statistically significant differences in demographic characteristics between class years in medical school Table 2.

**Table 1:** Demographic characteristics of study participants

Variable	Characteristic	Number	Percent
Gender	Female	84	50.60%
	Male	67	40.36%
	Nonbinary	2	1.20%
	Trans Male	1	0.60%
	Prefer Not To Answer	12	7.23%
Ethnicity	Asian Only	36	21.69%
	Black/African American Only	17	10.24%
	Latino/Latina Only	6	3.61%
	Multiracial	21	12.65%
	White Only	77	46.39%
	Prefer Not To Answer	9	5.42%
Year*	MS1	54	32.53%
	MS2	40	24.10%
	MS3	40	24.10%
	MS4	27	16.27%
	Prefer Not To Answer	5	3.01%
College Major	Both	13	7.83%
	Humanities	31	18.67%
	STEM	107	64.46%
	Prefer Not To Answer	15	9.04%

*PhD students are considered as MS2s. Research/scholarly year students between MS3 and MS4 are considered MS3s

**Table 2:** Comparing demographic characteristics by year in medical school

Variable	Characteristic	MS1	MS2	MS3	MS4	Total	*P*-value
Gender	Female	27 (56.2)	19 (54.3)	17 (54.8)	14 (56.0)	77	0.146
	Male	21 (43.8)	16 (45.7)	14 (45.2)	9 (36.0)	60	
	Nonbinary	0 (0.0)	0 (0.0)	0 (0.0)	2 (8.0)	2	
Ethnicity	Asian Only	14 (29.8)	8 (22.2)	10 (30.3)	4 (16.0)	36	0.478
	Black/African American Only	3 (6.4)	1 (2.8)	4 (12.1)	4 (16.0)	12	
	Hispanic Only	1 (2.1)	2 (5.6)	1 (3.0)	1 (4.0)	5	
	Multiracial	5 (10.6)	8 (22.2)	2 (6.1)	6 (24.0)	21	
	White Only	24 (51.1)	17 (47.2)	16 (48.5)	10 (40.0)	67	
College Major	Both	6 (12.8)	2 (5.9)	1 (3.3)	2 (8.0)	11	0.726
	Humanities	10 (21.3)	7 (20.6)	8 (26.7)	5 (20.0)	30	
	STEM	31 (66.0)	25 (73.5)	21 (70.0)	18 (72.0)	95	

Number (Percent)

### Historical knowledge

**Fig. 1:**
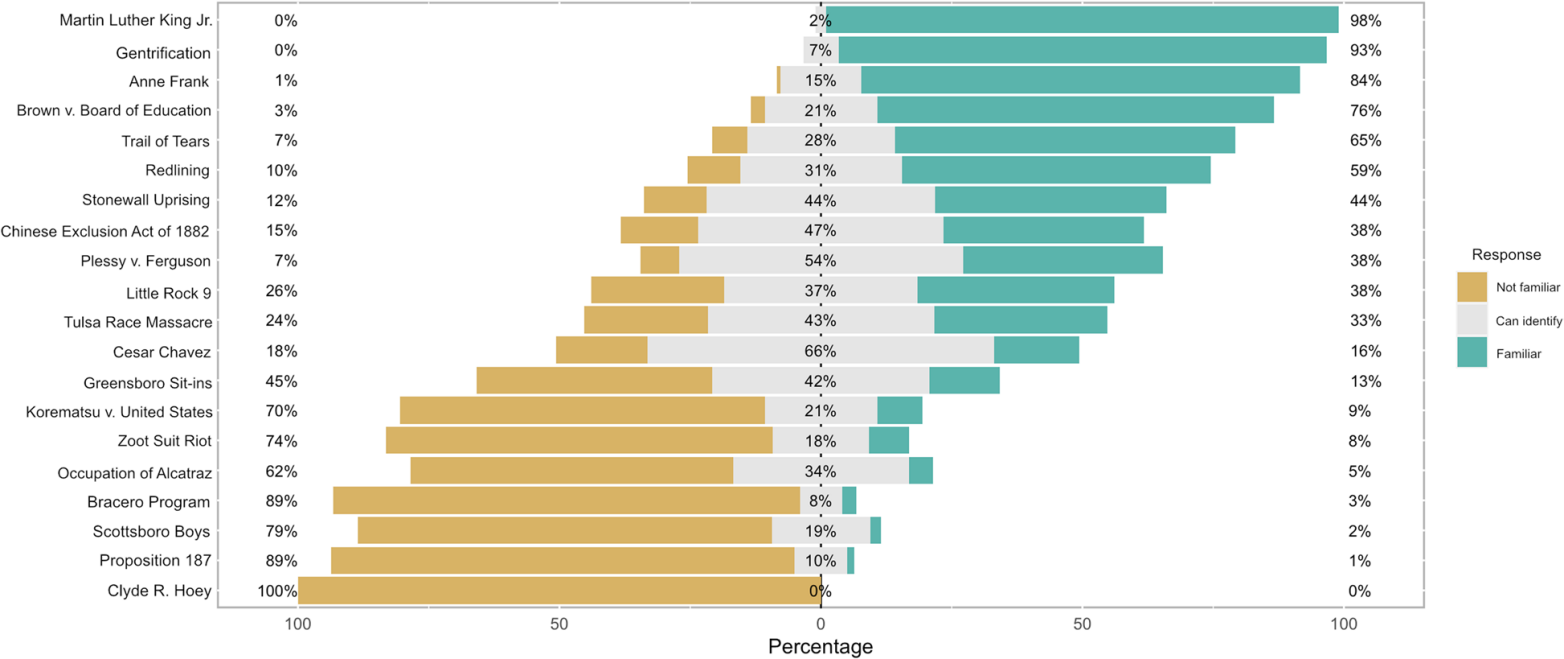
Self-rated knowledge of historical terms

Figure 1 describes the percentage of students who were familiar, could identify, or were not familiar with a variety of racially important historical events or persons. Martin Luther King Jr. and Clyde Hoey were used as controls for additional analyses; outlier responses were eliminated.

Individual historical racial variables significantly associated with medical school class included Scottsboro Boys (*p*=0.032) and César Chávez (*p*=0.043). Historical variables reflected a range of historical timepoints, racial groups, and terms and were further grouped into racial categories race as follows: African American History (Martin Luther King Jr., *Brown v. Board of Education*, *Plessy v. Ferguson*, Little Rock 9, Tulsa Race Massacre, Greensboro Sit-ins, and Scottsboro Boys); Asian American History (Chinese Exclusion Act of 1882, *Korematsu v. United States*); Latin American History (Cesar Chavez, Zoot Suit Riot, Bracero Program, Proposition 187); Native American History (Trail of Tears, Occupation of Alcatraz); and general racial historical terms (Gentrification, Redlining). Anne Frank and Stonewall Uprising were counted separately, as they each represent one example of their relevant history. On average, students were most familiar with general terms, African American, and Native American historical events, but not as familiar with Latin American and Asian American history Table 3. Mean scores in each category were not statistically significant with medical school class. Multiracial identity was significantly associated with increased knowledge of African American history (*p*<0.01).

**Table 3:** Historical knowledge among students, by year in medical school

	MS1	MS2	MS3	MS4	Total	*P*-value
**African American History**	2.2 (0.4)	2.2 (0.3)	2.2 (0.4)	2.2 (0.4)	2.2 (0.8)	0.904
**Asian American History**	1.7 (0.5)	1.8 (0.5)	1.9 (0.6)	1.8 (0.6)	1.8 (1.1)	0.414
**Latin American History**	1.5 (0.4)	1.4 (0.3)	1.6 (0.4)	1.4 (0.3)	1.5 (0.7)	0.170
**Native American History**	2.0 (0.4)	2.0 (0.5)	2.1 (0.4)	1.9 (0.5)	2.0 (0.9)	0.749
**General History**	2.7 (0.4)	2.7 (0.4)	2.8 (0.3)	2.8 (0.4)	2.8 (0.8)	0.930

Mean response from 1-3 (SD)

We accounted for possible confounding variables in a multivariate logistic regression model, adjusting for medical school year, college major, gender, and race. With respect to African American historical terms, participants identifying as Asian were less likely to be familiar when compared to other groups (OR=0.31, *p*=0.027). Humanities college majors were more likely to be familiar with terms regarding Latin American history as compared to STEM majors (OR=3.46, *p*=0.040).

### Advocacy statements

**Fig. 2:**
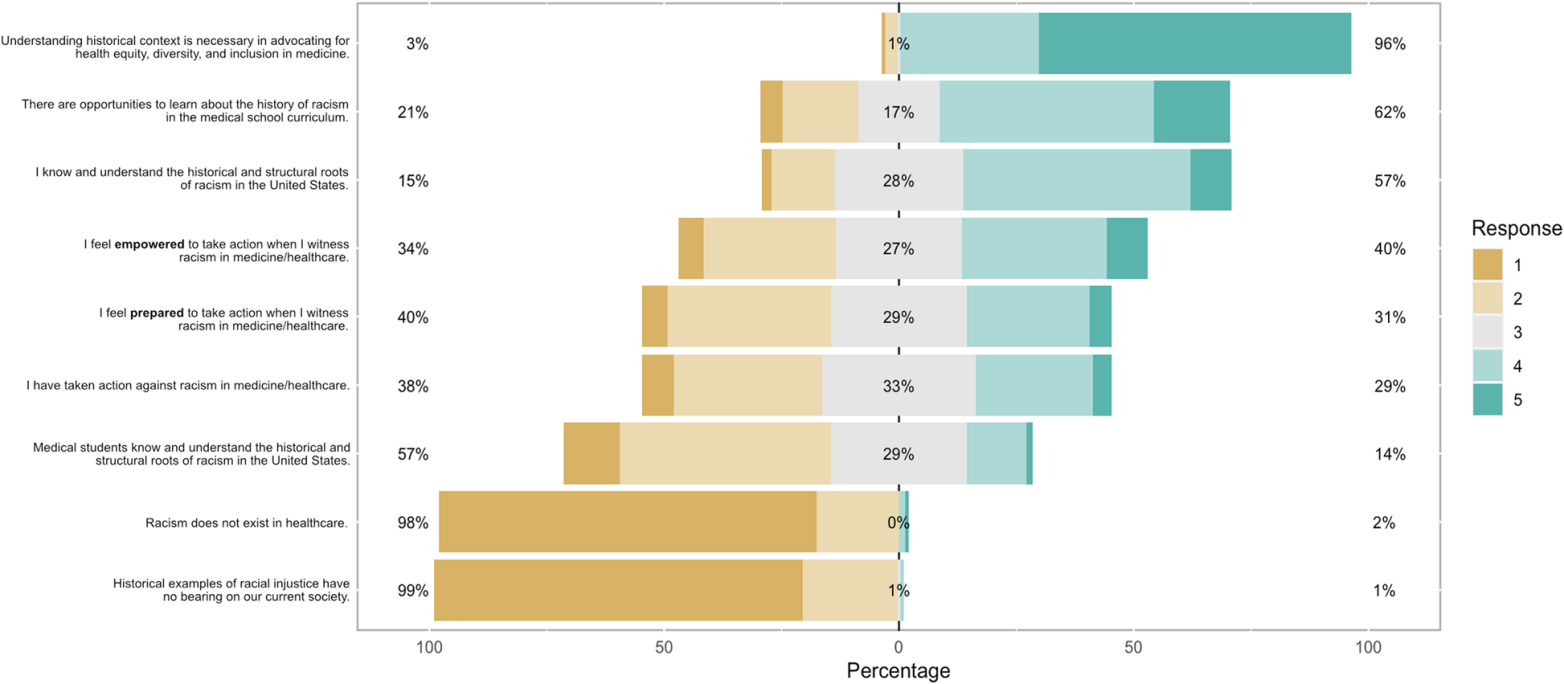
Agreement with statements regarding the role of health equity, education, and preparedness to act

Figure 2 describes the percentage of student responses to various advocacy statements, ranging from ‘I know the historical roots of racism’ to ‘racial justice has no bearing on medical school curriculum.’ No statistically significant differences were found between the current year in medical school and responses to these statements. Only a minority of students felt prepared (31%) or empowered (40%) to act, with no differences across years in medical school. 96% of students surveyed either agreed or strongly agreed to the statement “Understanding historical context is necessary in advocating for health equity, diversity, and inclusion in medicine.” Similarly, most students agreed that racism exists in medicine and that historical examples have a bearing on current society. Our analysis also examined the relationship between agreement of advocacy statements and mean historical knowledge. Students who had more historical knowledge of racial terms were significantly more likely to agree with the statements ‘I know the roots of racism’ (*p*<0.001) and ‘I have taken action’ (*p*<0.001).

## Discussion

Almost all (96%) students either agreed or strongly agreed to the statement “Understanding historical context is necessary in advocating for health equity, diversity, and inclusion in medicine.” Yet, students rated historical racial events, terms, or persons as “familiar” only 35% of the time, on average. Furthermore, 57% of students agreed or strongly agreed that they knew and understood the historical and structural roots of racism in the U.S., yet the same amount, 57%, expressed that they did not believe other medical students had this knowledge. This is consistent with our hypothesis. Therefore, there is a clear knowledge gap of important historical racial events present among medical students. When stratified by medical school class, there were very few significant statistical differences found between year and knowledge base. This suggests that students are not only entering medical school with a low knowledge base, but that they are also not learning new material regarding the experiences of historically oppressed communities during their time in medical school.

The historical events that most students were familiar with partially ranged depending on the racial category the term referenced. While almost all students were highly familiar with Martin Luther King Jr. (98%) and Anne Frank (84%), almost none knew about events such as Proposition 187 (3%) or the Bracero Program (1%), seminal events in Latin American history. Participants also tended to be familiar with larger trends of gentrification and redlining. Such terminology provides a common language for anti-racist and health equity work, which may explain the higher degree of familiarity among students. Knowledge of general terms and African American historical events appeared the highest, while knowledge of Latin American and Asian American history fell further behind. Considering the high prevalence of anti-Asian hate crimes during the COVID-19 pandemic [37] and the consequent health impacts among Asian American populations [38], medical schools should consider increasing Asian-American representation in the curriculum, including topics such as the tax of the model minority [39], which has profound implications for trainees, faculty, and patients alike. Similarly, there are myriad examples of unsanctioned, unethical medical experimentation on Latino patient populations [40] that are rarely explored in medical school curricula, as well as health implications of U.S. immigration policy [41–43]. Increasing such representation may improve anti-racist and health equity work with respect to other populations.

Statistical significance was found with multiracial identity and increased knowledge of African American history (*p*<0.01), and a humanities background with increased knowledge of Latin American history (*p*=0.017), but not other categories. These findings were not expected, and additional research should explore these relationships among identity, education in humanities, and historical knowledge. Furthermore, there were no significant statistical differences found between an individual’s identified race and their knowledge of events in that particular racial group. One might assume that an individual of a given race might be more familiar with historical events and terminology that affected their own race. Our research team was able to locate only one study regarding this relationship, which found no significant association with identifying as Black and knowledge about Black history [44]. This was consistent with our finding. However, Adams-Bass et al. found that the relationship between these two variables were mediated by racial socialization and self-esteem; namely, messages internalized from the media they consume and how these messages informed perception of one’s own racial identity. One troubling interpretation of our finding is that students attending medical school are becoming detached from their identities. It is indeed possible that the process of medical education itself may detach students from significant historical events and experiences affecting their own racial ancestry. This suggests a need to increase Latino, African American, and Asian American representation throughout curricula, starting even before medical school. In fact, the findings from the study by Adams-Bass et al. suggests that the more one is aware of Black history, the more likely one will have a high regard for Black people despite messages that present Black people negatively [44]. Together, our data supports the need for creating space for people from historically marginalized racial and ethnic groups to engage with historical events and experiences affecting their own racial group and the impact of those events on health equity.

Overall, only a minority of students felt prepared (31%) or empowered (40%) to act in the face of racial injustice in healthcare. Interestingly, MS3s felt less prepared to take action than other years of medical students. This may be because at our institution, MS3s move to clinical settings, where inherent power dynamics may emerge and grading may be more subjective. There could also be a disconnect between learning anti-racist practices and knowing how to tangibly advocate when one witnesses injustice. Reading and discussing anti-racist principles allow for meaningful engagement, but curricular efforts may fall short of real-world application. Practical application of such principles into advocacy and action, such as through simulation or other immersive methodologies, may prove revolutionary in bridging this disconnect. Additionally, medical schools have institutional policies around supporting students who chose to act, such as through antiretaliation policies, although future research can better elucidate to what extent such policies and hierarchy influences anti-racist action.

Of note, there was a significant association between mean historical knowledge and agreement with the advocacy statements “I know the roots of racism” (*p*<0.001) and “I have taken action” (*p*<0.001). Furthermore, increased knowledge of historical racial events was associated with an increased confidence knowing the roots of racism and in taking action to address racial injustice. This suggests that although only a minority of students have acted in the face of racial injustice, having a greater knowledge base of historical racial events can equip and empower students to do so. These findings suggest that increasing historical knowledge can have an important impact in improving health equity and racial injustice.

Our study faced several limitations. Our sample was a convenience sample drawn from a single, large urban medical school with a history of self-selection by individuals with an interest in and commitment towards social justice in medicine. In addition, sample size was small, and all survey responses were self-reported, which could lead to both selection bias and social desirability bias. It is possible that those who chose to participate were those more likely to be attuned to this work and even more knowledgeable than the full student body. In that case, our findings would be even more profound, as actual familiarity with racial history might be even lower. Despite the anonymity of responses, participants might also want to appear more knowledgeable and more supportive of anti-racist and health equity work than they might be. We tried to minimize this social desirability bias by including positive and negative controls. Despite efforts to increase representation of people from historically marginalized racial and ethnic groups in medicine over the past several years, our sample size was 46% White. Across U.S. medical schools, this group represents approximately 50% of all medical students [36]. While externally valid, the smaller sample size of underrepresented populations in medical schools limits interpretation of findings for these students. With larger representation, there might be increased historical knowledge, although examining individual racial groups in this study does not support this theory.

Overall, our data suggest that while medical students believe that understanding the historical context is important and that racism plays a substantial role in healthcare, they lack knowledge of key historical racial events. Increasing historical knowledge in medical school curricula or requiring students to enroll in a race-related history or similar course before matriculation could prove beneficial. Understanding the racial backdrop that large systems, such as the healthcare system, have been built on as well as the racial context that shapes the lived experiences of patients is necessary in advocating for health equity, diversity, and inclusion in medicine.

## Conclusion

This study demonstrates that while students agree that racism has no place in healthcare and knowing relevant history is essential, there remains a paucity of knowledge regarding many events and figures in the history of American race relations and civil rights, with implications for future physicians’ patient care and health equity efforts. These findings could inform the recommendation for an undergraduate requirement of studying race-related history and other relevant coursework before arriving at medical school. Additionally, continued efforts to integrate the historical context and broad consequences of racism in medicine into existing curricula may prove beneficial. Medical schools should continue to collect data on these important questions and study to what extent it informs health equity and anti-racist practices. While this study demonstrates a clear discordance between attitudes towards health equity and student knowledge, further research is needed to examine student perspectives more closely and further elucidate the factors that drive such discordance. Prospective studies examining student knowledge with the implementation of different education programs and modalities could also be beneficial. Future research could benefit from simulation methodologies where students can practice advocating and taking action in a safe, controlled setting. Then, additional study could evaluate how such training prepares students for practical application of such skills in real-world settings, such as an observational study on clinical wards, or in-situ simulation on clinical clerkships. Supporting the preparation and empowerment of students to speak up when they witness injustice in medicine is a critical area for improvement in medical education. Students approach medical school with complex identities; efforts to create space for reflection and engagement with these experiences and historical legacies of oppression may provide a safe way of connecting students with their own histories and empowering their future action.

## Supplementary Information

**Additional file 1.**

## Data Availability

The datasets generated and/or analyzed during the current study are not publicly available to protect the privacy of research participants, but deidentified data beyond those provided in this publication are available from the corresponding author on reasonable request.
