# Supplementary material for: Medical students’ knowledge of race-related history reveals areas for improvement in achieving health equity
Source: BMC Med Educ. 2022 Aug 10;22:612. doi: 10.1186/s12909-022-03650-x (PMC9365447; doi:10.1186/s12909-022-03650-x)
Supplement: Supplementary file 1 — Additional file 1. [file 12909_2022_3650_MOESM1_ESM.docx]

**Supplement A: Survey**

1. History Knowledge Questions

Below is a list of historical people, events, and concepts. Please indicate your familiarity with each of these terms on the following scale:

1. Do not recognize

2. Can identify, but uncertain or somewhat familiar with details/significance

3. Highly familiar with the term, details, and significance

Here is an example relating to President Abraham Lincoln:

1 = “Never heard of him”

2 = “He was president and is on the $5 bill”

3 = “He served as president during the United States Civil War until his assassination, issuing the Emancipation Proclamation and seeking to preserve the union.”

Of note, some of these terms may evoke disturbing associations. Please take this survey in a safe place and do not feel obliged to complete it.

Terms:

1. Scottsboro Boys
2. César Chávez
3. Martin Luther King Jr.
4. Gentrification
5. Little Rock 9
6. *Korematsu v. United States*
7. Proposition 187
8. *Plessy v. Ferguson*
9. Clyde R. Hoey
10. Bracero program
11. “Trail of Tears”
12. Chinese Exclusion Act of 1882
13. Redlining
14. Occupation of Alcatraz
15. Tulsa Race Massacre
16. Zoot Suit Riot
17. Greensboro Sit-ins
18. Stonewall Uprising
19. *Brown v. Board of Education*
20. Anne Frank

II. Advocacy Statements

Please indicate to what extent you agree or disagree with the following statements (5pt Likert Strongly Disagree to Strongly Agree):

1. Racism does not exist in healthcare.
2. Medical students know and understand the historical and structural roots of racism in the United States.
3. I know and understand the historical and structural roots of racism in the United States.
4. Understanding historical context is necessary in advocating for health equity, diversity, and inclusion in medicine.
5. Historical examples of racial injustice have no bearing on our current society.
6. There are opportunities to learn about the history of racism in the medical school curriculum.
7. I feel *prepared* to take action when I witness racism in medicine/healthcare.
8. I feel *empowered* to take action when I witness racism in medicine/healthcare.
9. I have taken action against racism in medicine/healthcare

III. Background Info/Demographics

1. What is your current year in medical school?
2. In what year did you start medical school?
3. What was your college major?
4. With what gender do you identify?
5. With what race(s) do you identify?
6. With what ethnicity(ies) do you identify?
